# Groin Pain Outcomes After Laparoscopic Transabdominal Preperitoneal Hernia Repair: A Prospective, Observational, Tertiary Care Center Study

**DOI:** 10.7759/cureus.113590

**Published:** 2026-07-29

**Authors:** Kuozokhotuo Suohu, Indu B Dubey, Harpyar Singh, Anurag Mishra, Nirupendra G T, Mayank Kushwaha, Ketan Banerjee, Rajat Dinkar

**Affiliations:** 1 Department of General Surgery, Vardhman Mahavir Medical College and Safdarjung Hospital, New Delhi, IND; 2 Department of Surgery, Vardhman Mahavir Medical College and Safdarjung Hospital, New Delhi, IND

**Keywords:** chronic postoperative pain, groin pain, inguinal hernia, laparoscopic hernia repair, tapp, visual analog scale

## Abstract

Background

Transabdominal preperitoneal (TAPP) repair is a widely adopted laparoscopic technique for inguinal hernia repair, offering advantages such as reduced postoperative pain and faster recovery. Because postoperative groin pain can adversely affect patient recovery and quality of life, evaluation of early pain outcomes following TAPP repair remains clinically important. This study aimed to evaluate the severity and progression of postoperative groin pain following elective TAPP repair for primary unilateral inguinal hernia using the Visual Analog Scale (VAS) and to assess postoperative recovery in terms of return to normal activity and analgesic requirements over a three-month follow-up period.

Methodology

This prospective observational study was conducted at the Department of General Surgery, Vardhman Mahavir Medical College and Safdarjung Hospital, New Delhi, over an 18-month period. In total, 41 patients with uncomplicated primary unilateral inguinal hernias underwent elective TAPP repair. Pain was assessed using the VAS on postoperative day one, day seven, month one, and month three. Time to return to normal daily activities and duration of analgesic use were also recorded.

Results

The mean age of the patients was 45.1 ± 16.43 years. The mean VAS score decreased significantly from 3.34 ± 0.89 on postoperative day one to 1.95 ± 0.59 on day seven and 0.8 ± 0.82 at three months (p < 0.001). All patients reported only mild pain from day seven onward, and no patient reported moderate or severe postoperative groin pain at the three-month follow-up. Most patients (56.5%) resumed normal daily activities within 8-14 days postoperatively. The mean duration of oral analgesic use was 5.6 ± 1.39 days, and the mean duration of clinically significant postoperative groin pain was 6.6 ± 2.7 days.

Conclusions

TAPP repair for primary unilateral inguinal hernia was associated with a favorable short-term postoperative pain profile, with a significant reduction in pain scores over the three-month follow-up period. Patients also demonstrated favorable short-term recovery, reflected by a relatively short duration of analgesic use and return to normal daily activities within approximately two weeks. Longer follow-up and larger comparative studies are required to evaluate chronic postoperative groin pain and further validate these findings.

## Introduction

Inguinal hernia repair remains one of the most commonly performed general surgical procedures worldwide [1]. With a lifetime risk estimated at 27-43% in men and 3-6% in women [2,3], inguinal hernias represent the most frequent type of abdominal wall hernia, necessitating effective and durable treatment strategies. Over the past few decades, laparoscopic techniques such as transabdominal preperitoneal (TAPP) and totally extraperitoneal repairs have gained prominence due to their association with reduced early postoperative pain, faster recovery, and lower recurrence rates compared to traditional open repairs [4].

Among long-term complications of inguinal hernia repair, chronic groin pain (CGP) has emerged as a critical factor affecting patient satisfaction and quality of life. Defined as pain persisting for more than three to six months postoperatively, CGP can lead to functional disability, impaired daily activities, and psychological distress [5,6]. The reported incidence of CGP ranges from 10% to 12%, with a subset experiencing moderate-to-severe pain impacting activities of daily living [7].

While TAPP repair has demonstrated benefits in minimizing acute postoperative pain, its role in mitigating chronic pain remains debated. Neuropathic mechanisms, such as intraoperative nerve injury or entrapment, and inflammatory responses to prosthetic mesh are among the proposed etiologies of persistent groin pain. Patients are increasingly concerned about groin pain rather than recurrence as the predominant factor affecting quality of life [8]. Given the variability in pain perception, definitions of CGP, and follow-up practices across studies, further evaluation is warranted.

This study aimed to assess the severity and progression of postoperative groin pain following elective TAPP repair for primary unilateral inguinal hernia using the Visual Analog Scale (VAS) at postoperative day one, day seven, month one, and month three. Secondary objectives included evaluation of postoperative analgesic requirements and time to return to normal daily activities.

## Materials and methods

Study design and setting

This prospective observational study was conducted in the Department of General Surgery, Vardhman Mahavir Medical College and Safdarjung Hospital, New Delhi, from August 2022 to January 2024.

Study participants

A total of 41 patients aged >18 years with uncomplicated primary unilateral inguinal hernia scheduled for elective TAPP repair were included.

Inclusion and exclusion criteria

Patients with uncomplicated primary unilateral inguinal hernia were included. Patients with immunocompromised status or comorbidities such as diabetic neuropathy were excluded.

Ethical considerations

Written informed consent was obtained from all participants before inclusion in the study. Study approval was obtained from Vardhman Mahavir Medical College and Safdarjung Hospital (approval number: IEC/VMMC/SJH/Thesis/2023-03/CC-135).

Surgical procedure

All procedures were performed under general anesthesia by the same senior laparoscopic surgeon using a standardized TAPP technique. Pneumoperitoneum was established using a 10-mm umbilical camera port and two 5-mm working ports. After creation of the peritoneal flap, the preperitoneal space was dissected, and the hernia sac was completely reduced.

A 15 × 10 cm Ultrapro™ lightweight partially absorbable composite mesh was placed to adequately cover the entire myopectineal orifice. Mesh fixation was performed using non-absorbable tackers, with an average of four tackers used per patient. Tackers were placed over Cooper’s ligament and the medial superior aspect of the mesh while carefully avoiding the triangle of pain and the triangle of doom to minimize the risk of nerve and vascular injury.

The peritoneal flap was closed over the mesh using continuous running barbed absorbable sutures (V-Loc™). The mean operative time was approximately 50 minutes. All procedures were performed by the same senior surgeon experienced in laparoscopic inguinal hernia repair, thereby ensuring a standardized operative technique throughout the study.

Postoperative analgesic protocol

All patients received a standardized postoperative analgesic regimen. Intravenous analgesics were administered during the immediate postoperative period according to institutional protocol, followed by oral non-steroidal anti-inflammatory drugs and/or paracetamol after discharge. Rescue analgesia was provided when clinically indicated. The duration of analgesic use recorded in this study refers to the number of days patients required oral analgesics following surgery.

Outcome measures

The primary outcome was severity of groin pain assessed using the VAS, a validated pain assessment tool, at predefined intervals. Secondary outcomes included duration of pain, duration of analgesic use, and time to return to normal daily activities.

Follow-up

Patients were discharged on postoperative day one and followed up at postoperative day seven, day 30, and three months.

Statistical analysis

Data were analyzed using SPSS version 29.0 (IBM Corp., Armonk, NY, USA). Quantitative variables were expressed as mean ± standard deviation (SD). Changes in postoperative pain scores over time were analyzed using repeated-measures analysis of variance (ANOVA). Changes in postoperative pain scores over time were analyzed using repeated-measures ANOVA. If the assumptions for repeated-measures ANOVA were not met, a non-parametric Friedman test would be considered. Qualitative variables were analyzed using the chi-square test or Fisher’s exact test, as appropriate. A p-value of <0.05 was considered statistically significant.

## Results

Study population and demographics

A total of 41 patients were included in the study. The mean age was 45.1 ± 16.43 years. The age distribution is shown in Table 1.

**Table 1:** Age distribution of patients.

Age group (years)	Number (n)	Percentage (%)
≤30	8	19.5
31–40	7	17.1
41–50	8	19.5
51–60	10	24.4
>60	8	19.6

Right-sided inguinal hernias were more common than left-sided hernias, accounting for 58.5% (n = 24) and 41.5% (n = 17) of cases, respectively. Right-sided indirect inguinal hernias were the most frequent subtype (31.7%, n = 13), followed by right-sided direct inguinal hernias (26.8%, n = 11) and left-sided direct inguinal hernias (26.8%, n = 11). Left-sided indirect inguinal hernias accounted for 14.6% (n = 6). No pantaloon or other hernia types were observed in the study population. The distribution of hernia laterality and type is summarized in Table 2.

**Table 2:** Distribution of hernia laterality and type.

Laterality	Hernia type	Number (n)	Percentage (%)
Left	Direct inguinal hernia	11	26.8
Left	Indirect inguinal hernia	6	14.6
Right	Direct inguinal hernia	11	26.8
Right	Indirect inguinal hernia	13	31.7
Total	Left-sided hernias	17	41.5
Total	Right-sided hernias	24	58.5

Postoperative pain assessment

A statistically significant reduction in pain over time was observed (repeated-measures ANOVA: F = 52.4, p < 0.001). The mean VAS scores are shown in Table 3.

**Table 3:** Mean VAS scores over time. Repeated-measures analysis of variance: F = 52.4, p < 0.001. VAS = Visual Analog Scale; POD = postoperative day

Time point	Mean VAS score ± SD
POD 1	3.34 ± 0.89
Day 7	1.95 ± 0.59
Month 1	1.95 ± 0.63
Month 3	0.8 ± 0.82

On postoperative day one, the majority of patients experienced mild pain (VAS 0-4), with only one patient reporting moderate pain. By postoperative day seven, all patients reported mild pain, and this trend continued at one month and three months. At the three-month follow-up, all patients had either no pain or mild pain, and none reported moderate or severe postoperative groin pain. The identical mean VAS scores at postoperative day seven and month one reflect rounding of the calculated mean values, although the distribution of individual patient scores differed between the two follow-up visits, as demonstrated by the differing standard deviations and VAS score distributions.

Distribution of pain scores

The distribution of VAS scores at different time intervals is shown in Table 4. There was a clear shift toward lower VAS scores over time, with 46% (n = 19) of patients reporting no pain at three months.

**Table 4:** Distribution of VAS scores. Chi-square test: χ² = 68.7, p < 0.001. VAS = Visual Analog Scale

VAS score	Day 1	Day 7	Month 1	Month 3
0	0	0	0	19 (46%)
1	0	7	10	12 (29%)
2	11	30	24	10 (25%)
3	7	3	7	0
4	22	1	0	0
5	1	0	0	0

Postoperative recovery outcomes

Duration of Pain

The mean duration of clinically significant postoperative groin pain was 6.60 ± 2.70 days. Duration of pain was defined as the period during which patients experienced continuous postoperative pain requiring regular analgesic medication. Overall, 73.2% (n = 30) of patients experienced pain for 3-7 days, while 26.8% (n = 11) experienced pain for 8-14 days. Mild residual discomfort recorded by low VAS scores at later follow-up visits was analyzed separately and was not included in the calculation of pain duration.

Analgesic Requirement

The mean duration of oral analgesic use was 5.60 ± 1.39 days. Overall, 87.8% (n = 36) required analgesics for three to seven days, whereas 12.2% (n = 5) required them for more than seven days.

Return to Normal Activity

The mean time to return to normal activity was 13.70 ± 2.91 days. Overall, 7% (n = 3) resumed activities within one week, 56.5% (n = 23) within 8-14 days, and 36.5% (n = 15) required more than 14 days. These findings are summarized in Table 5.

**Table 5:** Summary of recovery parameters.

Parameter	Mean ± SD
Duration of pain (days)	6.60 ± 2.70
Duration of analgesic use (days)	5.60 ± 1.39
Return to activity (days)	13.70 ± 2.91

## Discussion

This prospective observational study evaluated postoperative groin pain and recovery outcomes following TAPP repair for primary inguinal hernia. The findings demonstrate a progressive and statistically significant reduction in postoperative pain during the three-month follow-up period, along with early return to normal activity. At the three-month assessment, no patient reported moderate or severe postoperative groin pain.

The mean VAS score showed a marked decline from 3.34 ± 0.89 on postoperative day one to 0.8 ± 0.82 at three months, indicating effective pain control and favorable recovery. Notably, all patients reported only mild pain at three months, with 46% (n = 19) experiencing complete resolution of pain.

These findings are consistent with previous studies, including those by Tolver et al. [9], which demonstrated that postoperative pain following laparoscopic repair decreases significantly over time. Similarly, Koninger et al. [10] reported lower postoperative pain and lower rates of persistent pain following laparoscopic repair compared with open repair.

Aasvang and Kehlet [11] emphasized that chronic pain is a more significant determinant of quality of life than recurrence. Although no patient reported moderate or severe postoperative groin pain at the three-month follow-up, the present study was not designed to definitively assess chronic postoperative groin pain because follow-up ended at the earliest time point at which chronic pain begins to be evaluated.

This study has several strengths, including its prospective design, use of a standardized surgical technique performed by a single experienced team, and uniform pain assessment using a validated tool at predefined intervals. Additionally, the focus on pain as a primary outcome adds to the limited Indian literature in this domain.

This study has several limitations. First, the sample size was relatively small, and no a priori sample size calculation was performed, limiting statistical power to detect uncommon outcomes such as CGP. Second, this was a single-center observational study without a comparison group; therefore, comparative conclusions regarding TAPP versus other techniques cannot be drawn. Third, follow-up was limited to three months, which represents only the earliest threshold at which chronic postoperative groin pain begins to be assessed. Therefore, this study cannot definitively determine the incidence or absence of chronic postoperative groin pain, and its findings should be interpreted as reflecting short-term postoperative pain outcomes. Longer follow-up is required to accurately evaluate chronic pain after TAPP repair. Fourth, postoperative pain assessment relied solely on the VAS, which evaluates pain intensity but does not assess neuropathic symptoms, functional impairment, patient comfort, or health-related quality of life. In addition, several patient-related factors that may influence postoperative pain, including preoperative groin pain, previous lower abdominal surgery, psychiatric illness, chronic analgesic use, body mass index, smoking status, occupation, and physical activity level, were not systematically collected or analyzed. The absence of these variables may have resulted in residual confounding and should be addressed in future prospective studies. Finally, all procedures were performed by a single experienced surgeon at a single tertiary care center, which reduced operator-related variability but may limit the generalizability of the findings to other institutions or surgeons with different levels of experience.

## Conclusions

TAPP repair for primary inguinal hernia was associated with a favorable short-term postoperative pain profile in this prospective observational study, with a significant reduction in pain scores over the three-month follow-up period. At the three-month assessment, no patient reported moderate or severe postoperative groin pain. Patients undergoing TAPP repair also demonstrated favorable short-term recovery, reflected by a relatively short duration of analgesic use and return to normal daily activities within approximately two weeks. These findings suggest that TAPP repair is associated with encouraging short-term postoperative pain and recovery outcomes in this cohort. However, given the single-arm design, small sample size, and limited follow-up duration, larger prospective comparative studies with longer follow-up are required to evaluate long-term postoperative groin pain and to further validate these findings.
